# Systematic review of the diagnostic performance of serum markers of liver fibrosis in alcoholic liver disease

**DOI:** 10.1186/1476-5926-11-5

**Published:** 2012-12-28

**Authors:** Julie Parkes, Indra Neil Guha, Scott Harris, William MC Rosenberg, Paul J Roderick

**Affiliations:** 1 Primary Care & Population Sciences Faculty of Medicine University of Southampton (MP 805) South Academic Block Southampton General Hospital, Tremona Rd, Southampton, SO16 6YD, UK; 2 Nottingham Digestive Diseases Centre Biomedical Research Unit University of Nottingham, Nottingham, UK; 3 Institute for Liver and Digestive Health, University College London, Division of Medicine, London, UK; 4 UCLH/UCL NIHR Comprehensive Biomedical Research Centre, London, UK

**Keywords:** Alcoholic liver disease, Systematic review, Serum markers, Liver fibrosis

## Abstract

**Background:**

Alcoholic liver disease (ALD) is a significant cause of death and morbidity. Detection of liver fibrosis at an early stage could provide opportunities for more optimal management. Serum markers of liver fibrosis offer an alternative to biopsy. Evidence of the performance of biomarkers in ALD is needed and a systematic review to evaluate available studies was conducted.

**Methods:**

Electronic databases were searched. Studies were included if they evaluated paired samples of biopsy and serum, and presented data as sensitivity, specificity, or ROC curves.

**Results:**

15 studies were included- median participant number = 146 (range 44–1034). Studies differed with respect to patient populations. 6 single markers were evaluated (mostly Hyaluronic Acid), and ten combined panels. Biomarkers could discriminate between people with severe fibrosis/cirrhosis with high diagnostic accuracy- HA (median AUROC 0.79 range 0.69-0.93), panels (median AUROC 0.83 range 0.38-0.95). Significant heterogeneity precluded pooling. Performance was poorer for detecting less severe fibrosis.

**Conclusions:**

There are limited numbers of small studies evaluating the accuracy of biomarkers in identifying fibrosis on biopsy in ALD. Some showed promise (both HA alone and some panels) in the identification of cirrhosis/severe fibrosis and could be used to rule it out in heavy drinkers. Biomarkers less accurate with less severe fibrosis.

## Introduction

Alcohol related deaths are an important health concern worldwide. In the UK 85% of such deaths are due to cirrhosis and recent epidemiological studies have shown that although mortality rates from cirrhosis are falling in most countries absolute rates remain high, and in the UK and Eastern Europe the trend is upwards with 18% rise in deaths from alcohol related causes between 2000 and 2004 [1-5]. In these countries alcohol consumption is high and increasing and patterns of drinking have changed over the past three decades –binge drinking and a rise in hazardous drinking in younger women. Alcoholic Liver Disease (ALD) therefore represents a serious public health problem and is likely to get worse in the UK in the coming decades.

Clinicians and patients require accurate information about the degree of liver fibrosis in ALD to assess disease severity in order to predict outcome, guide management decisions and monitor disease. Detection of fibrosis in people drinking hazardously at an early stage or before clinical symptoms of hepatic decompensation could provide opportunities for more optimal management. This is a challenge in a disease process with few characteristic symptoms or signs. The current reference standard to ascertain the stage of fibrosis is histology obtained through liver biopsy. This is an invasive test and subject to limitations both in its acquisition (sampling error, length of biopsy, morbidity and mortality), subsequent analysis (intra and inter observer variability) and inherent drawbacks as a reference standard (ordinal categorical variable representing continuous biological process) [6-8]. In the past decade efforts have been made to find other tests to accurately evaluate fibrosis. Serum markers of liver fibrosis offer an attractive alternative to liver biopsy, as they are less invasive, may allow dynamic calibration of fibrosis, and are potentially more cost effective. Evidence of the diagnostic performance of such serum markers of liver fibrosis in Chronic Liver Disease are needed to assess the clinical utility and effectiveness of such tests in the diagnosis, prognosis and management of liver disease. Systematic reviews of the diagnostic performance of serum markers in chronic hepatitis C (CHC) and non alcoholic fatty liver disease (NAFLD) have been published but none so far on the evaluation of markers in ALD [9-13].

In order to provide such evidence, a systematic review was conducted to locate, collate, appraise and analyse studies that evaluated the performance of serum markers in the diagnosis of liver fibrosis in ALD.

## Methods

A systematic literature review was conducted following accepted published principles to ascertain the diagnostic performance of serum markers of liver fibrosis [14].

Sources searched included:

•Electronic databases 1980 – April 2009

•Cochrane Library 2009

•Reference lists from relevant articles

MEDLINE, EMBASE were searched using a search strategy derived from the literature (search strategy available from authors). Search terms were added following initial searches as appropriate.

No authors were contacted for further information.

### Inclusion/exclusion criteria

A serum marker was defined as any measure that could be derived from a blood sample

Studies were included if they;

•were systematic reviews, meta-analyses or primary studies of diagnostic tests

•were written in English

•used liver biopsy as a reference standard

•presented data as sensitivity or specificity or diagnostic accuracy or receiver operator characteristic curve (ROC) analyses

•included >30 participants (as smaller studies will be underpowered to produce precise estimates of test performance and would be more likely to produce zero denominator effects in a 2 × 2 table. Confidence intervals would be very wide and inclusion in SROC where studies are unweighted may result in skewed unreliable results).

•data were extractable by cause of liver disease and by fibrosis stage.

Studies were excluded if data were presented only in abstract form.

Studies identified by the search strategy were assessed for inclusion by two reviewers (JP and ING).

### Data extraction strategy

Data extraction was undertaken by one reviewer (JP) and checked by a second reviewer (ING) with any disagreements being resolved through discussion. A third reviewer (PR) was consulted to resolve persisting issues. Information collected included patient demographics, test assay details; background prevalence of fibrosis severity, risk factors, histological parameters, statistical methods used, and test performance characteristics consistent with columns in Tables 1 and 2.

**Table 1:** Characteristics of studies evaluating serum markers in alcoholic liver disease

**Study Author: Yr published (date of study when reported) Country*(No. centres)***	**Total no patients**	**Patient selection**	**Alcohol consumption inclusion criteria**	% cirrhosis (significant fibrosis*)	**Age Yr mean (SD)**	**% male**	**Liver biopsy scoring system**	**Serum marker or panel**
		**Recruitment details (where reported)**			**Fibrosis stages**		***Length biopsy/no portal triads***	
Gabrielli 1989 [15] Italy *(1)*	44	Patients with ALD on biopsy /clinical	n/r	n/r	52	84	Local *n/r*	PIIINP
		Consecutive prospective recruitment		fibrosis 29% no fibrosis 15%				
Poynard 1991 [16] (1982–1987) France *(1)*	624	Patients admitted with alcoholism or diagnosed ALD	≥ 50 g alcohol daily for last 5 years	29 (11)	49	75	Local *n/r*	PGA (GGT PT Apo A1)PT
	(a) 333 training			24% no fibrosis				
	(b) 291 validation	Consecutive prospective recruitment						
Li 1994 [17] USA *(1)*	44	Patients undergoing biopsy for clinical reason. with h/o heavy alcohol	>80 g daily at least 5 years	23 (70)	45 (range	100	Local *n/r*	PIIINP TIMP 1
		Prospective recruitment		Periven fibrosis 23%	227–69)			
Oberti 1997 [18] France*(1)*	160 total *(a)109 compensated*	Admissions for alcoholism/ diagnosed ALD	>50 g alcohol daily for 5 years with abn AST > 6 m	59	65	n/r	modified METAVIR *n/r*	HA
		Consecutive prospective recruitment						PT
Tran 2000 [19,20] (1997–1998)	146	Heavy drinkers admitted for detoxification+/−rehabilitation	>80 g alcohol daily for >5 year	40 (51)	49	73	Local *n/r*	HA
								PGA
								YKL
France*(1)*		Consecutive prospective recruitment						Tran index
								(HA; PT; Apo A1)
Plevris 2000 [21]	70	Patients with ALD diagnosed by histology	n/r	n/r	n/r	n/r	Local *n/r*	HA
		Prospective recruitment						
Croquet 2002 [22]	240	Patients admitted for alcoholism or ALD	50 g daily past 5 years	48 (74)	n/r	n/r	METAVIR	PT
							*20 ± 7 mm*	
							*(≥10 mm in 98% cases)*	
France*(1)*		Prospective recruitment					*12 ± 5 portal tracts*	
**Study Author: Yr published (date of study) country*No. centres***	**Total no patients**	**Patient selection**	**Alcohol consumption inclusion criteria**	% cirrhosis (significant fibrosis*)	**Age Yr mean (SD)**	**% male**	**Liver biopsy scoring system**	**Serum marker**
		**Recruitment details (where reported**					***Length biopsy/no portal triads***	
Stickel 2003 [23] Germany *(1)*	87	Admissions for alcohol withdrawal symptoms in current drinkers	>100 g alcohol daily	14 (44)	n/r	n/r	Local; Ludwig; Knodell *n/r*	HA
				Steatosis + mild fibrosis 23%				
				Steatosis + mod fibrosis + inflam 8%				
				Severe fib + inflam 30%				
Rosenberg 2004 [24] (1998–2000) *England (8) Germany Italy Sweden*	64	Patients with excess alcohol consumption history and histology	Assessed by each centre	27	44	63	Scheuer	ELF panel
							Ishak	(HA TIMP1 PIIINP age)
		Consecutive prospective recruitment					*≥12 mm ≥5 portal tracts*	

Naveau 2005 [25] (1996–2000) France(1)	221	Patients with active history of excess alcohol consumption admitted to hospital (24% decompensated cirrhosis) and with available histology	>50 g alcohol daily for 1 year	31(64)	47	77	METAVIR	Fibrotest (α2M, apoA1, bilirubin, GGT, haptogloblin, corrected for age + sex)
				Stage 0 7%			Mean length 15 mm ± 05	
				Stage 1 329%				
				Stage 2 22%			Frags = 2.2 ± 0.1	
		Prospective recruitment		Stage 3 11%			portal tr 14.4 ± 0.7	HA
				Stage 4 31%				
Cales 2005 [26] (1994–2002) France *(1)*	95	Heavy drinkers with ALD on histology	>50 g daily >5 years	41 (80)	49.8 (11.2)	71.6	METAVIR	Fibrometer (PT α2M HA)
		Consecutive prospective recruitment		Stage 0 13%			*Median Length 18.4 ±6.0*	
				Stage 1 18%				
				Stage 2 17%				
				Stage 3 12%				
				Stage 4 41%				
Lieber 2006 [27] USA *(23)*	1034: (a) 507 pre-cirrhotic (b) 527 decompensated cirrhosis	Patients with heavy alcohol consumption + fibrosis/cirrhosis on biopsy/clinical in 2 treatment RCTs	80 g ethanol daily >5 years HCV negative	51(66)	(a) 51	98	Ishak	APRI
					(b) 56		*n/r*	(AST Platelets)
		Prospective recruitment						
**Study Author: Yr published (date of study) country*No. centres***	**Total no patients**	**Patient selectionrecruitment details (where reported)**	**Alcohol consumption inclusion criteria**	% cirrhosis (significant fibrosis*)	**Age Yr mean (SD)**	**% male**	**Liver biopsy scoring system**	**Serum marker**
							***Mean length mm/no portal tracts***	
Nguyen –Khac 2008 [28]	103	Patients with attending hepato-GI, alcoholism & Int Med depts. who were HBV- and HCV- without decompensated cirrhosis who agreed to have liver biopsy	>50 g daily alcohol for >5 yrs	33 (75)	53 (9.6)	74	METAVIR	HA
				Stage 0 8%			*length 12.2 ±3 mm*	Hepascore
				Stage 1 18%			*Portal tracts 7.8 ± 2.7*	(bilirubin GGT HA age,sex α2M)
				Stage 2 23%				
				Stage 3 19%				PGA
		Prospective recruitment		Stage 4 32%				PGAA (PT GGT α2M, apoA1)
								APRI(AST Pl)
								Fibrotest
								Fibrometer
								*(fibroscan)
Lieber 2008 [29] (1994–2000)	247	Heavy alcohol consumption and fibrosis on biopsy	≥80 g daily . ≥5 years (~16 drinks daily for mean 19 yrs)	(45)	50	98	Worner & Lieber *n/r*	HA
				Mild fibrosis 55%				TIMP1
								P3NP
Naveau 2009 [30] (1996–2000 same population as 2005 paper) France (1)	218	Heavy alcohol consumption and available liver biopsy HCV- HIV-	≥50 g alcohol daily for1previous year	31 (63)	47 (0.07)	78	METAVIR	FT
				Stage 0 7%			Biopsy/ serum ≤1 month apart	Fibrometer (HA PT α2M)
		Retrospective		Stage 1 30%				
				Stage 2 22%			Mean length 15 mm ± 05	Hepascore (α2M GGT Bilirubin HA)
				Stage 3 10%			No frags 2.2 ± 0.1portal tr 14.4 ± 0.7	
				Stage 4 31%				Forns (age GGT cholesterol pl)
								APRI
								FIB4 (platelets ALT AST)

*(Significant fibrosis METAVIR stages 2-4: Ishak 3-6).

**Table 2:** Diagnostic performance of single markers

**Degree of fibrosis tested**	**Study**	**No.**	**AUC**	**Cut off used**	**Sens**	**Spec**	**PPV**	**NPV**	**LR + (95% CI)**	**LR – (95% CI)**
**HA**										
Cirrhosis	Oberti [18] (1997)	109*	n/r	60mcg/l	100	60	78	97	2.5 (1.7,3.6)	0.02(0.004,0.18)
	Tran [19] (2000)	146	n/r	60mcg/l	100	86	83	99	6.8 (4.1,11.4)	0.02 (0.004,0.1)
	Plevris [21] (2000)	70	n/r	100mcg/l	87	89	n/a	n/a	8.0	0.15
	Stickel [23] (2003)	87	**0.78**	250mcg/l	100	69	35	98	3 (2.0, 4.28)	0.10 (0.02,0.69)
	Naveau [25] (2005)	221	**0.93** (0.91,0.95)	n/r	n/r	n/r	n/r	n/r	n/r	n/r
	Nguyen-Khac [28] (2008)	103	**0.80** (0.68,0.92_	n/r	n/r	n/r	n/r	n/r	n/r	n/r
Stage 012 vs34	Stickel [23] (2003)	87	**0.76**	55.5 mcg/l	83	69	67	83	3(1.7, 4.2)	0.26 (0.13,0.53)
	Nguyen-Khac [28] (2008)	103 -	0.83 (0.74-0.92)							
	Lieber [29] (2008)	247	**0.69**							
F01vs 234	Naveau [25] (2005)	221	**0.79** (0.76-0.82)	n/r	n/r	n/r	n/r	n/r	n/r	
	Nguyen-Khac [28] (2008)	103	**0.80** (0.70-0.92)	n/r	n/r	n/r	n/r	n/r	n/r	n/r
**Degree of Fibrosis tested**	**Study**	**No.**	**AUC (95%CI)**	**Cut off used**	**Sens**	**Spec**	**PPV**	**NPV**	**LR + (95% CI)**	**LR-(95% CI)**
F0 vs 1-4	Nguyen-Khac [28] (2008)	103	**0.76** (0.58-0.94)	n/r	n/r	n/r	n/r	n/r	n/r	n/r
**P3NP**										
F012 vs34	Gabrielli [15] (1989)	44	n/r	16 ng/ml	71	50	n/r	n/r	1.4	0.6
	Lieber [29] (2008)	247	**0.67**							
F0 vs F1-6	Gabriella [15] (1989)	44	n/r	16 ng/ml	90	59	n/r	n/r	2	0.2
	Li [17] (1994)	44	**0.80** ±0.07	1.1 U/ml	45	100	94	44	6.8 (0.99, 47)	0.6 (0.42, 0.82)
Prothrombin Index**										
Cirrhosis	Oberti [18] (1997)	109	n/r	85%	n/r	n/r	n/r	n/r	n/r	n/r
	Croquet [22] (2002)	240	n/r	80%	81	99	99	85	101(14.3,713.5	0.2 (0.13,0.28)
	Tran [19] (2000)	146	n/r	85%	83	93	89	89	12.1(5.56,26.5)	0.2 (0.1,0.33)
**TIMP1**										
F012 vs 34(advanced fibrosis)	Lieber [29] (2008)	247	**0.68**		n/r	n/r	n/r	n/r	n/r	n/r
Any fibrosis (1994)	Li [17]	44	**0.96** ±0.03	313 ng/ml	n/r	n/r	n/r	n/r	n/r	n/r
**YKL**										
Cirrhosis	Tran [19] (2000)	146	n/r	330mcg/l	51	89	75	74	5 (2.4,8.6)	0.5 (0.4,0.7)
**ApoA1**										
Cirrhosis	Tran [19] (2000)	146	n/r	1.2 g/l	83	93	89	89	12.1 (5.6,26.5)	0.18 (0.10,0.33)

### Data analysis/synthesis

Data are presented with full tabulation of results of included studies.

Where data were available, 2 × 2 tables were constructed to derive sensitivity, specificity, predictive values, likelihood ratios (LR) and diagnostic odds ratios (DOR) at each threshold value. (Accepted levels for robust tests are - LR = <0.1, and + LR = >10, >5 and <0.2 give strong diagnostic evidence. For DOR reasonable test performances would be >30). Severity of fibrosis was defined by authors (for locally derived classifications) and as mild = stages 0,1, moderate/ severe stages 2–4, severe fibrosis stages 3,4 and cirrhosis stage 4 for those using METAVIR/Scheuer classifications.

## Results

The electronic search yielded 463 abstracts which were read in full. 41 full papers were retrieved of which 26 were excluded leaving 15 studies in separate populations to be included in the review (see Table 2). Reasons for exclusion were (may be >1 /study);

•Not primary study (editorial/non systematic review) n = 3

•Outcome was not fibrosis (usually alcoholic hepatitis) n = 6

•Participants <30 n = 1

•No results separable for ALD alone n = 6

•No results reported as sensitivity, specificity, ROC curves, diagnostic accuracy n = 11 (Most of these studies reported correlation coefficients/differences in means of serum markers between group with fibrosis and those with less fibrosis).

•No results for fibrosis alone separable from data that combined steatosis with fibrosis or fibrosis/cirrhosis with acute alcoholic hepatitis (AH) n = 4

No systematic reviews or meta-analyses were identified. Studies were conducted between 1989 and 2009. Study characteristics are shown in Table 2. The median age of participants in included studies was 50 years (range 44–65 years), 77% were male (range 63-100%) and the median number of study participants was 146 (range 44–1034). The median background prevalence of serious fibrosis/cirrhosis was 41% (14-59%). All of the studies were conducted in secondary/tertiary settings.

There was marked differences between the studies. Different scoring systems were used: METAVIR (or modified METAVIR) n = 6; Scheuer n = 1; Ishak n = 2; Knodell n = 1; Worner /Lieber n = 1, and locally generated n = 5 (mostly dividing fibrosis into mild, moderate or severe). 13/15 studies presented data that showed the performance of the markers in identifying cirrhosis/severe fibrosis (METAVIR stages 4 /3,4), 5/15 reported significant fibrosis (METAVIR stages 2–4), and 3/15 studies reported information identifying any fibrosis). All of the studies evaluated performance of markers using cross sectional data for paired samples of histology and serum. 14/15 studies recruited prospectively, and half recruited consecutive patients. There was heterogeneity of patient selection. Although all participants were recruited in a hospital setting, some were hospitalized and some were out- patients. There were also differences in both in the inclusion criteria and daily alcohol consumption. Inclusion criteria reported were patients with previously diagnosed ALD, and or “alcoholism” or heavy alcohol consumption, or patients admitted for rehabilitation/detoxification/alcohol withdrawal symptoms. The daily consumption of alcohol (where reported) varied with 1 study recruiting patients drinking >100 g of alcohol/day, 4 studies >80 g, and 6 studies >50 g, Inclusion criteria used a varied number of years drinking at these levels (range 5–10 years) reported, with one study having a mean alcohol consumption of 225 g/day for a mean of 19 years [29] (See Table 2). Some studies used the same population of patients with ALD to report the performance of different serum markers- single and panel tests- in two publications [25,30]. Another research group reported two studies which also used the same patient population, with the earlier study reporting results from 109 patients with compensated ALD recruited in 1994–95 and the later study adding further patients from 1997–98 and reporting from the whole cohort (n = 240) [2,18]. Both studies were included as data reported were different, with the earlier study reporting the performance of two serum markers and the later study having more participating patients but reporting results for one marker. This may reflect the difficulty in recruiting and retaining patients with this liver disease

The significant heterogeneity precluded pooling of results. Results are presented separately for single markers (Table 2) and for marker panels (Table 3) in the identification of cirrhosis (F4 METAVIR) cirrhosis, /severe fibrosis (F3/F4 METAVIR) and ‘significant’ fibrosis (F2-4-Metavir). There were 13 separate markers evaluated- 6 as single markers, and the remaining as components of 10 panels. 5/6 of those reported as single markers were also used in the panels. Three studies reported sensitivity and specificity at more than one threshold [25-27].

**Table 3:** Panel marker tests measuring fibrosis in ALD

**Fibrosis grade**	**Study**	**No.**	**Test**	**AUROCS**	**Cut off**	**Sens**	**Spec**	**PPV**	**NPV**	**LR+**	**-LR**
										**(95% CI)**	**(95% CI)**
Cirrhosis	Poynard [16] 1991	624	PGA	n/r	6	85	85	70	93	5.6 (4.5 7.01)	0.18 (0.12,0.25)
Cirrhosis	Tran [19] 2000	146	Tran	n/r		76	99	98	86	66.8 (9.5,471.2)	0.24 (0.15,0.37)
Cirrhosis	Naveau [25] 2005	221	Fibrotest	**0.95 (0.94, 0.96)**	0.3	84	41	39	85	1.4 (1.2,1.7)	0.39 (0.2,0.70)
					0.7	60	72	49	80	2.1 (1.6,2.9)	0.55 (0.40,0.75)
Cirrhosis	Lieber [27] 2006	1034	APRI	**0.79**	>2.0	17	86	56	50	1.2 (0.9,1.6)	1.0 (0.92,1.02)
Cirrhosis	Nguyen –Khac [28] 2008	103	Fibrotest	**0.84 (0.72,0.97)**	n/r	n/r	n/r	n/r	n/r	n/r	n/r
			Fibrometer	**0.85 (0.74,0.96)**	n/r	n/r	n/r	n/r	n/r	n/r	n/r
			Hepascore	**0.76 (0.63,0.90)**	n/r	n/r	n/r	n/r	n/r	n/r	n/r
			APRI	**0.56 (0.38,0.73)**	n/r	n/r	n/r	n/r	n/r	n/r	n/r
			PGA	**0.89 (0.82 0.97)**	n/r	n/r	n/r	n/r	n/r	n/r	n/r
			PGAA	**0.83 (0.73-0.93)**	n/r	n/r	n/r	n/r	n/r	n/r	n/r
Cirrhosis	Naveau [30] 2009	218	Fibrotest	**0.94 (0.90,0.96)**	0.56	90	n/r	n/r	n/r	n/r	n/r
					0.78	n/r	90	n/r	n/r	n/r	n/r
					>0.30	100	50	47	100	2.0	0.50
					>0.70	87	86	73	94	6.2	0.16
			Fibrometer	**0.94 (0.90,0.97)**	0.92	90	n/r	n/r	n/r	n/r	n/r
					0.997	n/r	90	n/r	n/r	n/r	n/r
					>0.50	99	62	54	99	2.6	0.38
					>1.0	88	88	76	94	7.3	0.14
			Hepascore	**0.92 (0.87,0.97)**	0.97	90	n/r	n/r	n/r	n/r	n/r
					0.99	n/r	90	n/r	n/r	n/r	n/r
			Forns	**0.38 (0.27,0.47)**	n/r	n/r	n/r	n/r	n/r	n/r	n/r
			APRI	**0.67 (0.59,0.75)**	n/r	n/r	n/r	n/r	n/r	n/r	n/r
			FIB4	**0.80 (0.72,0.86)**	n/r	n/r	n/r	n/r	n/r	n/r	n/r
F012vs 34 Severe	Rosenberg [24] 2004	64	ELF	**0.94 (0.84, 1.00)**	0.087	100	17	75	100	1.2 (1.1, 1.4)	0.06 (0.01, 0.3)
					0.431	93	100	100	86	68 (37,124)	0.08 (0.05,0.1)
F012vs 34 Severe	Nguyen –Khac [28] 2008	103	FT	**0.80 (0.7,0.9)**	n/r	n/r	n/r	n/r	n/r	n/r	n/r
			Fibrometer	**0.88 (0.8,0.95)**	n/r	n/r	n/r	n/r	n/r	n/r	n/r
			Hepascore	**0.83 (0.74,0.93)**	n/r	n/r	n/r	n/r	n/r	n/r	n/r
			APRI	**0.43 (0.30,0.56)**	n/r	n/r	n/r	n/r	n/r	n/r	n/r
			PGA	**0.84 (0.74 0.94)**	n/r	n/r	n/r	n/r	n/r	n/r	n/r
F012vs 34 Severe	Lieber [29] 2008	247	HA	n/r	n/r	76	68	53	86	2.4	0.35
			P3NP								
			TIMP1								
			Age								
			As panel								
F01 vs 2-4 Mod/severe	Cales [26] 2005	95	Fibrometer	**0.96 (0.94, 0.98)**	n/r	92	93	99	76	18 (2.7,125)	0.08 (0.2)
F01vs 2-4 Mod-severe	Naveau [22] 2005	221	Fibrotest	**0.84 (0.81 0.87)**	0.3	84	66	81	70	2.5 (1.8,3.4)	0.25 (0.16,0.40)
					0.7	55	93	93	54	7.4 (3.3,16.1)	0.5 (0.4,0.6)
F01vs2-4 Mod severe	Lieber [27] 2006	507	APRI	**0.70**	0.2	94	26	71	68	1.3 (1.2,1.4)	0.24 (0.17,0.33)
					0.6	47	82	84	44	2.6 (2.0,3.3)	0.65 (0.6,0.71)
					1.0	21	90	80	37	2.1 (1.5, 3.0)	0.88 (0.83,0.92)
					1.6	13	95	83	36	2.5 (1.5,4.1)	0.92 (0.88,0.95)
					2.0	9	97	86	35	3.1 (1.6,6.1)	0.94 (0.91,0.96)
F01vs2-4 Mod severe	Nguyen –Khac [28] 2008	103	Fibrotest	**0.79 (0.69,0.90)**		n/r	n/r	n/r	n/r	n/r	n/r
			Fibrometer	**0.82 (0.72,0.93)**		n/r	n/r	n/r	n/r	n/r	n/r
			Hepascore	**0.76 (0.64,0.88)**		n/r	n/r	n/r	n/r	n/r	n/r
			APRI	**0.54 (0.4-0.68)**		n/r	n/r	n/r	n/r	n/r	n/r
			PGA	**0.78 (0.68,0.89)**		n/r	n/r	n/r	n/r	n/r	n/r
			PGAA	**0.81 (0.71,0.91)**		n/r	n/r	n/r	n/r	n/r	n/r
F01vs2-4 Mod severe	Naveau [30] 2009	218	Fibrotest	**0.83 (0.77,0.88)**	0.23	90	n/r	n/r	n/r	n/r	n/r
					0.64	n/r	90	n/r	n/r	n/r	n/r
					>0.30	88	52	76	72	1.8	0.55
					>0.70	43	97	96	50	14.3	0.07
			Fibrometer	**0.83 (0.77,0.87)**	0.11	90	n/r	n/r	n/r	n/r	n/r
					0.95	n/r	90	n/r	n/r	n/r	n/r
					>0.50	74	74	83	62	2.85	0.35
					1.0	55	95	95	55	11.0	0.09
			Hepascore	**0.83 (0.77,0.88)**	0.25	90	n/r	n/r	n/r	n/r	n/r
					0.94	n/r	90	n/r	n/r	n/r	n/r
			Forns	**0.38 (0.30,0.46)**	n/r	n/r	n/r	n/r	n/r	n/r	n/r
			APRI	**0.59 (0.51,0.67)**	n/r	n/r	n/r	n/r	n/r	n/r	n/r
			FIB4	**0.70 (0.62,0.76)**	n/r	n/r	n/r	n/r	n/r	n/r	n/r
Mild fibrosis	Lieber [29] 2008	247	HA	n/r	n/r	74	76	86	53	3.1	0.34
			P3NP								
			TIMP1								
			Age								
			As panel test								
Any fibrosis	Nguyen –Khac [28] 2008	103	Fibrotest	**0.77 (0.63,0.90)**	n/r	n/r	n/r	n/r	n/r	n/r	n/r
			Fibrometer	**0.72 (0.57,0.87)**							
			Hepascore	**0.70 (0.51,0.89)**							
			APRI	**0.76 (0.58,0.95)**							
			PGA	**0.66 (0.50,0.82)**							
			PGAA	**0.74 (0.60,0.88)**							

### Single markers

All single markers studies were heterogeneous with respect to the grade of fibrosis identified by the test, and the thresholds reported (Table 2)*.*

i) Hyaluronic Acid (HA)

The most commonly measured single marker was HA (7 studies, total n = 1360), The studies were all small (n = ~200) and where reported different thresholds of HA concentration for positive test results were used (range 55 mcg/l - 250 mcg/l). Not all studies gave sufficient detail of analytical methods used to determine HA, but there were differences in methods used in those that did report the assay-a radiometric binding protein assay (used by three included studies); an enzyme linked binding protein assay, and immunoassay using a magnetic particle separation technique (2 studies). The inclusion criteria with respect to alcohol consumption were different for each study (>100 g alcohol daily; >80 g for >5 years, , >50 g daily alcohol for >5 yrs, >50 g alcohol daily for 1 year) as was the size of the studies (range n = 70-247). The severity of serious fibrosis varied between studies, with prevalence of cirrhosis in one study [22] being less than half that in the other studies

Seven studies evaluated its performance in the identification of cirrhosis or cirrhosis /severe fibrosis although only 4 of these reported AUROC values. One study reported results for the identification of patients with no or mild fibrosis. The AUROCs for the 3 studies identifying cirrhosis were discrepant −0.78, 0.80 and 0.93. The median AUC for predicting severe fibrosis/cirrhosis =0.79 (range 0.69-0.93). Overall the LRs and predictive values showed that HA was better at excluding cirrhosis/ severe fibrosis than detecting it, with NPVs consistently high ~90% for cirrhosis.

There are two direct comparisons of a panel and HA. These showed differing results. In the larger study [25] there was no significant difference between panel (Fibrotest) and HA at both identifying cirrhosis and moderate /severe fibrosis. In the other study [28] most of the panel tests had greater AUC values in predicting cirrhosis than HA alone (but 95% CI were overlapping) but at lower levels of fibrosis the performance of HA and panels are more similar. Overall HA was better at identifying cirrhosis alone than moderate/severe fibrosis (AUROC ~ 0.80) or milder fibrosis.

ii) Other single markers

There were more limited data on five other single markers, with only three studies presenting AUROC analyses. Prothrombin index had high LR + and predictive values in the identification of cirrhosis in two studies. One study reported performance of TIMP1 and PIIINP in the same population of patients as single markers and as part of a panel. The study found that the AUROC values were lower than in other studies of the same markers [29]. However this study population differed from the other studies in having a very high alcohol consumption over a long period of time

### Marker panels

**Figure 1:**
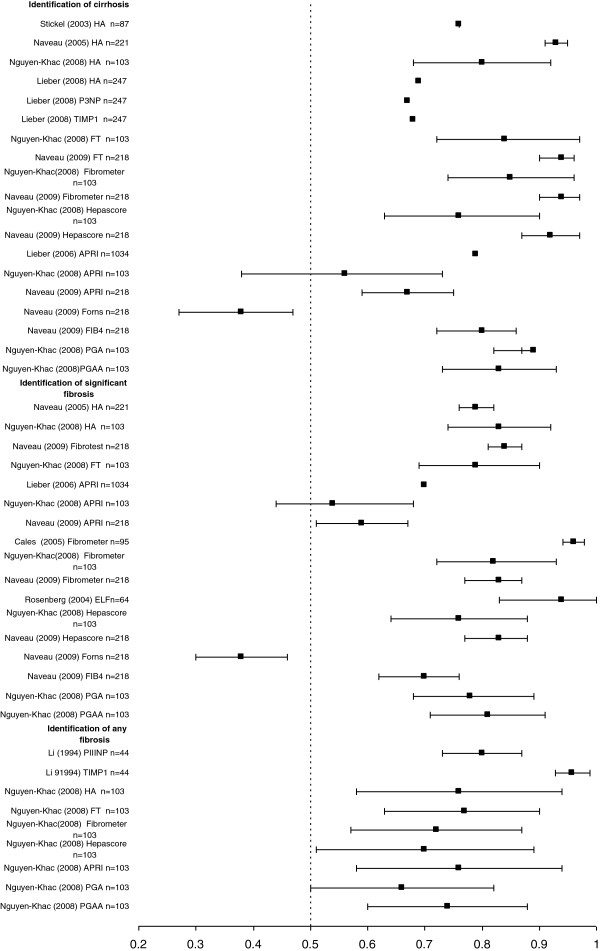
**Summary figure of the AUC results for serum markers in ALD in the identification of cirrhosis, significant fibrosis (2–4) and any fibrosis.** AUC values (where reported) for all serum markers studies in patients with ALD identifying cirrhosis, significant fibrosis or any fibrosis with 95% CI (where reported). Most studies are small (wide confidence intervals), varying in threshold reported, and where >1 study, per serum marker results are inconsistent.

Cirrhosis/severe fibrosis (Figure 1, Table 3). Eight studies assessed the performance in detecting cirrhosis/severe fibrosis, five of which reported AUROCs. Four studies were external validations of previously derived panels [25,27-30]. Several panels (Fibrotest, Fibrometer, Hepascore, ELF) showed promise in detection of cirrhosis with AUROCs >0.9, although one was small (ELF n = 64), and one showed no statistically significant difference to HA in direct comparison (Fibrotest). Common components of these panels are HA (in 3 panels), alpha macroglobulin (in 2 panels), GGT (in 2 panels). One panel (Tran index) reported a very high specificity and PPV compared to other panels. Simpler panels with ≤3components (for example PGA- Prothrombin Index, GGT and Apolipoprotein A1) performed as well as more complex panels –in a direct comparison AUROCs for cirrhosis PGA 0.89 Vs Fibrotest 0.84 Vs Hepascore 0.76, and for severe fibrosis/cirrhosis AUROCs PGA 0.84 Vs Fibrotest 0.80 Vs Hepascore 0.83 although this was only in one small study [25].

(ii) Moderate /severe fibrosis (Biopsy stages 2–4) The performance of eight panels were reported of which three had AUROCs >0.8 in detection of moderate/severe fibrosis, Three studies reported results for Fibrometer, with a varying range of AUROCs (0.96, 0.83, 0.82, total patients n = 416). Fibrotest AUROCs were 0.84,0.83, 0.79) (total n = 324); and it was not significantly more accurate than HA alone in direct comparison). Two studies reported results for Hepascore (AUCs 0.76, 0.83) total n = 321. Other panels had poorer performance in detecting moderately severe fibrosis.

Three studies reported results for APRI [24,25,27] ( AUCs 0.70, 0.54 0.59) total n = 828) and Forns index (AUC 0.38 95% CI 0.30,0.46). Those panel test external evaluations performed by groups other than the original authors showed a lower diagnostic performance. In general, panels of markers reported lower diagnostic performance in the detection of lesser stages of fibrosis than in cirrhosis [25,27-30].

## Discussion

A systematic review of the diagnostic performance of serum markers in identifying liver fibrosis on biopsy in patients with ALD using standard methodology found 15 primary studies. The evaluations used 13 different markers, for single markers most commonly HA (n = 7), and 10 marker panels. Serum markers were able to identify those people with severe fibrosis/cirrhosis with reasonable diagnostic accuracy (based on AUROCs). HA as a single marker performed well in identifying cirrhosis, as do some panels of markers. The performance of the serum markers was poorer at identifying lower grades of fibrosis, although few studies evaluated this. The paucity of the literature precluded further conclusions and summative analysis was not possible due to study heterogeneity.

The evidence base for serum markers in ALD lags behind that of Hepatitis C and non alcoholic fatty liver disease. The studies are fewer in number, have fewer participants, vary considerably in inclusion criteria, and have a higher prevalence of cirrhosis/severe fibrosis than in similar studies in Hepatitis C and NAFLD. They also tend to be older studies than other liver disease aetiologies, being less informed by recent advances in the rigour and standardisation required from design and reporting of diagnostic studies [31]. More recent studies have evaluated panels (two of which were external validation studies). Panels varied in their individual constituents, and in the number of components. Generally the values of AUROCs of panel tests in patients with ALD in predicting cirrhosis /sever fibrosis are comparable with those in NAFLD or Hepatitis C. For example in a metaanalysis of Fibrotest in Hepatitis C the mean AUROC for predicting significant fibrosis was reported as 0.77 (95% CI 0.75, 0.79) and in NAFLD 0.81 (95% CI 0.74 0.86) [2], and a summary AUROC for cirrhosis 0.82 [32]. Certain panels such as APRI seem to perform less well in ALD than in Hepatitis C. Summary AUROC for significant fibrosis was reported as 0.76 (95% CI 0.74 0.79) and for cirrhosis 0.82 (95% CI 0.79 0.86) [33,34].

There have been reports in the literature of the effect of current heavy alcohol consumption on circulating serum markers which may limit their performance in identifying the chronic effect of alcohol on fibrosis in patients who may be current drinkers. The mode of action of alcohol on the markers is unclear. Animal models have shown that alcohol may have an effect on serum markers such as HA in several ways- by alteration of communication between liver cells thereby affecting HA clearance and by direct effect on induction of hepatic sinusoidal endothelial cell dysfunction [35,36], Studies have shown that some markers are more susceptible to influences of acute consumption but results are not consistent. One study reported that some markers are affected (tenascin, laminin), some are unaffected (PIIINP, TIMP1), and some very variable (HA) [37]. One small study reported that mean levels of PIIINP but not TIMP1 rise with abstinence [38]. This confirmed the results from an earlier study which showed similar effect of alcohol on PIIINP [38] Direct studies of effects of alcohol on serum markers in clinical studies involve very small numbers and few studies have reported in the last 5 years. Most alcohol status (were reported ) is self report with some studies using collateral evidence when available. The included studies in this review did not all report current drinking status in detail. In 4 studies included patients were in-patients for alcohol withdrawal /rehabilitation, in 2 studies the patients were not abstinent. More data from large robust studies are needed to properly evaluate the influence of current alcohol intake (ideally quantified with objective measures/triangulated evidence) on markers, reporting results in terms of level of alcohol consumption and time of abstinence.

A major concern in drawing overall conclusions from this review is the considerable heterogeneity of the study populations. Whilst all included studies recruited patients from specialist clinics in secondary or tertiary settings (there were no studies set in primary care), there was variation in the population characteristics, such as level of alcohol consumption, and differences in the prevalence of severe fibrosis. This may lead to spectrum bias influencing diagnostic performance and additionally, affect generalisability. Design of the studies differed with variation in recruitment methods and inclusion criteria. All patients had to have had a biopsy (from inclusion criteria) which could introduce verification bias compared to those patients with excess alcohol consumption not selected for biopsy having a different disease severity than those who were selected. Only four studies reported any parameters by which biopsy quality could be judged, and half of these reported findings stratified by biopsy quality. Even when the tests were similar between studies, the thresholds used were different or not reported. Direct comparison between studies was made more difficult by the use of a range of fibrosis staging systems, largely locally generated. There was heterogeneity and lack of standardization of analytical methods used for the markers measurements and as these different assays may not be well correlated, external validity may be reduced and the determination of a single generalisable threshold remains problematic for those markers assayed locally. Access and availability of serum markers using commercial automated platforms may address this issue. There was incomplete reporting of co-morbidities and diagnostic test results, making appraisal and summative assessment difficult. The paucity of studies which looked at direct comparisons between panels, and between single marker and panels make it difficult to say one panel is more accurate than another. It is clear from this systematic review that the current serum markers are promising, improving and may provide additional diagnostic information in the identification and management of people with ALD.

The limitations of this review include lack of data to perform summative analyses and a focus on the ability of diagnostic tests to identify fibrosis alone. Detection of inflammation has not been addressed. Issues of spectrum bias which may have an impact on performance characteristics of the tests making direct comparisons between studies problematic, and this has not been directly addressed in this review. This is due to several main problems in accounting for such as bias. The first is a lack of a universally accepted system of dealing with this issue, especially in this group of patients with ALD. There have been some methodological suggestions published by one group in chronic Hepatitis C [39], who have used this method in a study in ALD patients [30]. Authors used standard population of same prevalence for all fibrosis stages and currently it is unclear if this has external validity or international acceptance by professionals working in this field. In addition the studies included in this review are older, use different classification systems for histology and have inconsistent and incomplete reporting of the individual stages of study participants. All of this makes accounting for spectrum bias problematic, complex and of questionable validity in this review. However it is an important issue and should be borne in mind when looking at results between studies.

### Clinical implications

For preventing and managing ALD it is important to identify those patients who are drinking hazardously and have clinically silent severe fibrosis/cirrhosis in order to focus interventions, to begin to screen for varices and Hepatocellular carcinoma or to prepare for possible liver transplant. Data presented in this review suggest that marker panels could be used effectively in this situation. It would be clinically useful to patients and clinicians to identify the proportion of hazardous drinkers who have developed liver disease to monitor disease progress more closely and to offer an opportunity for strategies aimed at reduction/abstention. Repeated serum marker measurement showing rise or decline in results may have an impact on lifestyle choices again allowing scope for reduction in alcohol consumption. These are speculative ideas and require further research. This group of patients often has erratic attendance at outpatient and biopsy appointments and may present in settings where invasive tests are inappropriate/difficult (e g prison). Access to non-invasive tests of liver fibrosis would be useful in the management of such patients.

### Future research

Large studies of patients with ALD need to be designed which can directly compare and validate in external populations, performance of existing markers, the identification of new markers or enhancement of existing tests to identify any, mild or moderate fibrosis. For example, methods such as proteomics and metabonomics may identify markers that can be incorporated into existing or new panels of markers, either in isolation or in combination with quantitative imaging techniques (such as elastography). This process might be facilitated by establishing an international reference library and quality assurance scheme. The evaluation of diagnostic performance should be accompanied by parallel evaluation of test performance for properties such as reproducibility, stability and linearity. Further work is needed to ascertain the diagnostic performance of markers in primary care setting. The limitations of liver biopsy may create a glass ceiling for potential non-invasive tests, and future studies should consider use of clinical outcomes as the reference standard. The few studies that have been reported in the literature on performance of serum markers in ALD predicting clinical outcomes rather than fibrosis have shown good performance for some panels of serum markers [27]. Fibrotest, Hepascore and Fibrometer A has been shown to be able to predict liver related mortality at 5 years and 10 years (AUC = 0.79 (95% CI 0.68,0.86) 0.77(95% CI 0.69,0.85) 0.80(95% CI 0.71,0.87) respectively, at least as well as biopsy (AUC 0.77 (95% CI 0.70,0.83). Forns index, APRI and FIB 4 had lower performance in predicting liver related mortality -AUCs 0.40 (95% CI 0.30,0.49), 0.60 (95% CI 0.50,0.69), 0.65 (95% CI 0.54 0.74 respectively. In a smaller population of patients with ALD the predictive performance of the ELF test has also shown AUC 0.80 (95% CI 0.70, 0.89) for liver related morbidity/mortality at 7 years (personal communication with Authors). Additional larger studies that can evaluate and compare performance of non invasive methods in predicting clinical outcomes in patients with ALD are needed.

In summary, none of the serum markers reported so far in the literature appear to have a very good performance for fibrosis severity less than moderate/severe fibrosis/cirrhosis. In general, performance decreases as severity of fibrosis being identified/ruled out decreases. HA shows some promise as a single marker in ruling out cirrhosis and to an extent severe fibrosis, but it is hard to know what threshold to use. Other single markers have less good performance when used alone. Some Panels (Fibrometer, Fibrotest Hepascore, and ELF) show promise in diagnosing cirrhosis/severe fibrosis but studies in ALD have small numbers.

## Conclusion

A systematic evaluation of the evidence of the diagnostic performance of serum markers of fibrosis in ALD has shown that there are few small studies published which show that serum markers are able to identify cirrhosis/severe fibrosis with good diagnostic accuracy, although study heterogeneity in design and outcome precludes pooling. In clinical practice, this may allow earlier exclusion of liver damage in hazardous drinkers permitting earlier and targeted interventions. The limitations of the liver biopsy may create a glass ceiling for potential non-invasive tests, and in this regard more studies using clinical outcomes should be evaluated.

## Competing interests

Professor William Rosenberg has received honararia for lecturing from Siemens Diagnostics.

## Authors’ contributions

JP and ING conducted the literature search and data extraction; SH participated in design and construction of quantitative display of data synthesis and provided statistical support, PJR and WR conceived of the study, participated in the design of the study, provided additional resource for literature search and study selection, and helped draft manuscript. All authors read and approved the final manuscript.
